# The Story of the Insane from Year to Year

**Published:** 1900-02-03

**Authors:** 


### THE STORY OF THE INSANE FROM YEAR TO YEAR.

Twelfth Series.

### WEST RIDING ASYLUM AT WAKEFIELD.

On January 1st this asylum contained 1,420 patients, and at the end of December there was an increase of 3 only. There were 270 first admissions, 72 readmissions, 145 recoveries, 42 discharged relieved or not improved, and 152 deaths. The recoveries were 42·39 per cent. of the admissions, and the deaths were 10·72 of the average number resident. Both of these percentages are higher than the present asylum average. Of the deaths 67 were caused by cerebral and spinal diseases, and of this total not fewer than 36 were caused by general paralysis. Four were from bronchitis, 8 from pneumonia, 18 from consumption, 1 from colitis, and 2 from enteric fever. Various moral causes, chiefly domestic trouble and mental anxiety, account for the mental disease in 53 of the admissions; and of the physical causes hereditary influence heads the list with 121, and intemperance in drink follows close on with 75, while sunstroke brings up the rear with 1. There were 6 cases of typhoid fever, and of these 2 died. Dr. Lewis attributes the outbreak to the disturbance of some old soil when getting out foundations for a new block of closets, and this is not an improbable cause, for it is well-known that the typhoid germ may retain its vitality for long periods. There were also three cases of ulcerative colitis. We have the fine work on this disease by Dr. Gemmel and Dr. Goodliffe, of Lancaster Moor Asylum, but it would be interesting to have Dr. Lewis' views also. A hospital for acute cases of insanity has been built, and is being most elaborately fitted up. An expectant asylum world awaits the results. Dr. Lewis deplores the small number of attendants who go to the nursing lectures conducted by Dr. Hearder, and certainly his remarks are not a bit too strong. The asylum is again full, and no further enlargement should be thought of. Another asylum is the only solution of the difficulty. There are among the patients 75 Catholics. What proportion of these may be fit to attend Mass it is impossible to say, but a priest attends at the asylum every Sunday. The Commissioners in Lunacy think this priest should be paid for his services, and they add that "the authorities do not hesitate to receive services gratis, whilst they would scorn to make use of a person's time and talents in any other asylum work without adequate remuneration." This is rather strong language to use to one of the most liberal committees in England, and we should like to know whether the committee asked the priest to attend the asylum. It is much more likely that he attends of his own accord, and if so he has no claim to a salary. Moreover, can the Commissioners point to any Catholic country where Protestant clergymen are allowed to attend public institutions gratuitously or otherwise? The committee say that "the management of the institution is very creditable to Dr. Bevan Lewis." The weekly cost per head is 9s. 6d.

